# Clinical Outcomes of First Metatarsophalangeal Joint Arthrodesis Using the BOFAS Registry: A Prospective Cohort Study

**DOI:** 10.1002/jfa2.70084

**Published:** 2025-09-30

**Authors:** Ferozkhan Jadhakhan, Makwana Nilesh, Mason Lyndon, Halliwell Paul, Rushton Alison

**Affiliations:** ^1^ College of Life Sciences Faculty of Health Education and Life Sciences Birmingham City University Birmingham UK; ^2^ Orthopaedic Surgery The Robert Jones and Agnes Hunt Orthopaedic Hospital NHS Foundation Trust Oswestry UK; ^3^ Trauma and Orthopaedic Department Liverpool University Hospitals NHS Foundation Trust Liverpool UK; ^4^ Department of Trauma and Orthopaedic Surgery Royal Surrey County Hospital NHS Trust Guildford UK; ^5^ School of Physical Therapy Faculty of Health Sciences Western University London Ontario Canada; ^6^ Centre of Precision Rehabilitation for Spinal Pain School of Sport Exercise and Rehabilitation Sciences University of Birmingham Birmingham UK

**Keywords:** clinical outcomes, first metatarsophalangeal joint (MTPJ) arthrodesis pathway, manchester‐oxford foot questionnaire, patient reported outcome measures, the british orthopaedic foot and ankle society (BOFAS) registry

## Abstract

**Background:**

This study investigated the quality and clinical outcomes of the British Orthopaedic Foot and Ankle Society (BOFAS) registry first metatarsophalangeal joint (MTPJ) arthrodesis pathway.

**Methods:**

A prospective cohort study using data derived from the BOFAS registry. Adults aged ≥ 18 years with a record of undergoing first MTPJ arthrodesis in the UK from 29/08/2014 to 31/10/2019. The pre‐ and post‐treatment pathway was evaluated by analysing Patient Reported Outcome Measures (PROMs) at baseline, 6 months and 12 months intervals. Consistency of data capture and completeness were explored using means, SD, medians and IQR for continuous variables and frequencies for categorical variables.

**Results:**

The mean age of the study population (*n* = 459) was 64.1 (± 12.1) years and 98.9% of the study cohort were female. Completeness of data collection was low for some items (e.g., medication 46%, surgical procedures 52%). Baseline completion of PROMs was moderate with 52.5% of participants providing MOXFQ (Manchester–Oxford Foot Questionnaire) pain and walking/standing scores at baseline. However, follow‐up response rates declined substantially to 27.2% at 6 months and 15.7% at 12 months. Improvement in PROMs by 12 months following surgery was statistically and clinically significant (*p* < 0.001), with median scores of 10 [IQR: 0–20] for MOXFQ pain, 5.5 [0–21] for walking/standing, 0 [0–19] for social interaction, 5 [1–31] for NRS pain and 0.8 [0.7–1.0] for EQ‐5D‐5L.

**Conclusion:**

The analysis highlights the clinical benefits of first metatarsophalangeal joint (first MTPJ) fusion surgery, with improvements in pain intensity, walking/standing ability, social interaction and quality of life. The BOFAS registry serves as a valuable tool for collecting patient‐reported outcome measure (PROM) data, providing important insights into treatment effectiveness and participant well‐being. Strengthening the data collection capabilities of the BOFAS registry could further enhance our understanding of the benefits of first MTPJ fusion and inform future treatment strategies.

## 1 Introduction

Health outcome data are increasingly being used to improve quality of care to ensure cost effectiveness and patient‐centred care. Data registries as a source of data have proliferated, enabling specialist members to examine participant outcomes to afford evidence on optimal treatment. In the UK, National Joint Registries for arthroplasty have collected close to 2 million records since 2003, enabling surgeon and implant review and increasing participant safety [1] A central registry for foot and ankle surgery was proposed by the British Orthopaedic Foot and Ankle Society (BOFAS) [2], and in 2014 the BOFAS registry was established and commenced data collection. The registry is open to the members of the society, who use it voluntarily without mandatory completion. Since inception, the registry has seen increasing data being collected nationally. The aim of the BOFAS registry is to help demonstrate the effectiveness of treatment, assess performance to reduce variation, help understand participants' expectations, support patient centred care and provide data for future research. In addition, it helps surgeons track their outcomes for participants and allows for comparison locally and nationally. Confidence in the data is central to any evaluation of the data.

The BOFAS registry [2] outcome data encompass: EuroQol‐5 Dimension (EQ‐5D), Manchester–Oxford Foot Questionnaire (MOXFQ) and Numeric Rating Scale (NRS), along with demographic (e.g., age) and clinical data (e.g., prescription drugs). The Patient Reported Outcome Measures (PROMs) enable clinicians to assess participants' post‐operative health status and quality of life associated with healthcare or treatment and formulate care tailored to participant needs [3]. On inception of the registry, first metatarsophalangeal joint (MTPJ) and ankle arthrodesis were selected as the procedures to commence initial data collection, due to the belief that there would be minor national variability in their undertaking. Unlike joint arthroplasty, many foot and ankle procedures have significant variability in their surgical techniques; for example, over 150 procedures are described for treatment of hallux valgus [4].

Arthrodesis is a commonly performed surgical procedure for the treatment of arthritis of the first MTPJ [5, 6]. Other indications for first MTPJ Joint arthrodesis include hallux valgus, failed cheilectomy, resection arthroplasty, or implant arthroplasty and hallux valgus accompanied by significant functional disability and pain [7]. Hallux rigidus (osteoarthritis) affects 1 in 45 people over the age of 60 years, with a strong preponderance for females [8]. Associated pain can lead to significant disability [9]. The resulting economic burden includes direct costs (health care use/cost) and indirect costs (e.g., work‐days loss due to disability, productivity). The overall costs resulting from osteoarthritis of any joint include loss of economic production of over £3.2 billion; with £43 million spent on community services and £215 million on social services [10]. The level of disability also has the potential to increase the predisposition of affected individuals to associated co‐morbidities [11, 12]. Previous studies have shown that BMI was associated with increasing severity of pain and disability in OA of other joints [13] and foot pain [14]. These may be substantial and so a successful surgical outcome is important in saving valuable health system resources. Evidence suggests that First MTPJ arthrodesis can significantly improve quality of life regarding disability, function and pain [15]. Most existing studies are based on individual surgeon practice or small institutional cohorts. Registry data, such as those collected by BOFAS, have the potential to provide broader insights into patient‐reported outcomes at a national level. However, for registry data to be meaningfully interpreted and used in clinical decision‐making, the consistency, completeness, and performance of the registry itself must also be evaluated. This study represents the first attempt to assess the PROM‐based outcomes of first MTPJ arthrodesis and, in doing so, provide insight into the utility and limitations of the BOFAS registry.

## 2 Aim

To evaluate patient‐reported outcomes following first MTPJ arthrodesis using BOFAS registry data, and to assess the consistency and completeness of the registry.

## 3 Objectives

1. To evaluate pre‐ and post‐surgery PROMs and associated clinical with the first MTPJ arthrodesis using data from the BOFAS registry
2. To explore post‐operative (poor vs. good) outcome following surgery
3. To examine the consistency of the data.
4. To assess the completeness of data capture.

## 4 Methods

### 4.1 Design

A prospective cohort study was conducted using data derived from the BOFAS registry. While the registry itself collects data prospectively at defined time points (pre‐operative, 6 months and 12 months post‐operative). All available records of first MTPJ fusion procedures recorded in the registry up to the point of data extraction were considered. However, only cases with pre‐operative PROMs were included in the analysis to allow for comparison with post‐operative outcomes. The quality of the study design and reporting was assured using the Strengthening the Observational Report on Epidemiology (STROBE) guidelines [16].

### 4.2 Data Source

The dataset was obtained from the BOFAS registry database. The registry is designed for online, electronic input of Patient Reported Outcome Measure and clinical data. Participants have the flexibility to either complete the questionnaires electronically in clinic on a departmental tablet/PC or by email.

### 4.3 Participant Eligibility Criteria

Adults aged ≥ 18 years with a record of a first MTPJ Arthrodesis BOFAS registry pathway commencement in the UK from 29/08/2014 to 31/10/2019 (these data do not include all MTPJ arthrodesis as it is voluntary to upload data). Following listing for surgery, participating surgeons offer their participants the option to participate in the registry data collection and are consented at this point. Participants are requested to provide an email address as part of the consenting process. Prior to the scheduled operation, participants are asked to fill in a pre‐operative questionnaire, and then again after 6 months and a year post surgery. Participants can withdraw from the registry at any point. Personal details such as name, address, telephone number and email address are captured, with participant's consent, in addition to details of diagnosis, operation and any complications. However, no personal data were shared as part of this project, and data were managed/analysed in an anonymised format. A total of 459 participants were eligible for inclusion based on having available baseline PROMs data. However, due to the voluntary nature of the registry, some variables were incomplete. For example, medication data were available for 213 participants (46%), and smoking status was recorded for 137 participants (30%). Although all eligible participants were analysed, the completeness of specific data fields varied. Consequently, while all 459 participants met the eligibility criteria, participation and completeness of data varied across different measures and time points.

### 4.4 Ethics

Ethical approval was obtained from the University of Birmingham Research Ethics Committee (ERN_19‐1274AP2). All participants gave informed consent for their data to be used in research and evaluation when they registered with the BOFAS registry. Therefore, no additional consent was required for this study, as the data were collected during routine clinical care. Participants provide consent for their data to be used for evaluation purposes when they initially sign up to the BOFAS registry.

### 4.5 Variables

The data items collected in BOFAS include demographic data, diagnosis, procedure, complications, and outcome data including PROMs: Manchester–Oxford Foot Questionnaire (MOX‐FQ), Euro‐Qol EQ‐5D‐5L, NRS and the EQ‐VAS‐health questionnaire pre‐surgery and at 6 and 12 post‐operatively. The MOXFQ is a validated 16‐item questionnaire designed to assess foot and ankle health across three domains: pain, walking/standing, and social interaction. Each domain is scored separately, with scores transformed to a 0–100 scale, where a higher score indicates worse symptoms or disability [17]. The EQ‐5D‐5L is a generic health‐related quality of life instrument consisting of five dimensions (mobility, self‐care, usual activities, pain/discomfort, and anxiety/depression), each rated on five levels of severity. It produces a health utility index, where higher scores indicate better health status [27]. The EQ‐VAS is a 0–100 visual analogue scale where participants rate their overall health, with 0 representing the worst health imaginable and 100 the best [28]. The NRS was used to assess pain intensity, with scores ranging from 0 (no pain) to 100 (worst pain imaginable), where higher scores indicate greater pain severity [29].

### 4.6 Management of Missing Data

Variables such as BMI, smoking ROMS were missing from the BOFAS dataset. An additional missing stage category was included in the stratified analysis, for example, (BMI < 20, ≥ 20 < 30, ≥ 30 < 40, ≥ 40, Missing), to describe the level of completeness of the data. Missing data occurred primarily because some participants did not complete specific sections of the baseline or follow‐up questionnaires, rather than due to a failure to attempt data collection. PROMs data were incomplete at follow‐up intervals (particularly at 6 and 12 months), rather than entirely missing at baseline, and these gaps have been accounted for in the analysis.

### 4.7 Statistical Analysis

#### 4.7.1 Descriptive Analysis of Demographics and Clinical Data (Objectives 1 and 2)

For descriptive analyses, means, SD, medians and IQR for continuous variables and frequencies for categorical variables were calculated. Variability of distribution for each variable was tested separately. For data with high skewness, distribution was tested using histograms, and medians and IQR used to describe the central tendency and variability of data.

#### 4.7.2 Post‐Operative (Poor vs. Good) Outcome Following Surgery (Objective 3)

Pre‐operative and 12 months follow‐up PROMs scores were assessed using the Wilcoxon signed‐rank test to compare paired samples (scores before and 12 months after surgery) in the same participant group as data were not normally distributed and medians are a more robust indicator of central tendency. The Wilcoxon signed‐rank test was used to determine whether there is a statistically significant difference in the median of good outcome versus poor outcome. Good outcome is defined as a PROM score lower at 12 months compared to baseline (baseline score minus 12 months score) in the same participant group.

#### 4.7.3 Pre‐ and Post‐Clinical Outcomes (Objective 4)

Descriptive statistics were calculated for PROMS at each interval (baseline and follow‐up) and differences reported as percentages. Continuous variable data were reported as median and IQR. As the recorded PROMs data were not normally distributed, the analysis used nonparametric statistics. Data were grouped into the three domains of the MOX‐FQ (pain, walking/standing and social interaction). Median baseline and post‐operative scores (6 and 12 months) were calculated separately. These scores are presented independently on a scale of 0–100: 0 represents the best possible health state and 100 the worst [17]. Boxplots were used to compare medians and IQR for PROM scores at baseline, 6‐ and 12‐month intervals. The Wilcoxon signed‐rank test was used to compare the paired samples (e.g., scores before and 12 months after surgery).

## 5 Results

### 5.1 Characteristics of Study Population

The study population consists of *n* = 459 participants. A descriptive analysis of the baseline demographics, clinical data, health related quality of life, functional status and pain physical/functional outcome are provided in Table 1. The mean age of the study population was 64.1 ± 12.1 years. Approximately 50% of the participants were over the age of 65 at the time of surgery and 98.9% of the study cohort were female. Most participants reported undergoing a primary procedure (93.8%). Participants were more likely to have a BMI of 25–29.5 kg/m^2^ and less likely to be a nonsmoker (80.3%). However, these figures are based on available data only, as BMI and smoking status were not recorded for all participants, and this should be considered when interpreting the findings.

**TABLE 1:** Characteristics of the first MTPJ arthrodesis population at baseline (excluding missing data).

Variables	*n* = 459	
Age (yr.), mean (SD)	64.1+/−(12.1)	
Age, no. (%)	No. (%)	Cumulative (%)
< 40	11 (2.4)	2.4
40–44.9	13 (2.8)	5.2
45–49.5	26 (5.7)	10.9
50–54.5	35 (7.6)	18.5
55–59.5	50 (10.9)	29.4
60–64.5	70 (15.2)	44.6
65–69.5	81 (17.6)	62.2
70–74.5	86 (18.7)	80.9
≥ 75	87 (18.9)	99.8
Female, no. (%)	284 (98.9)	
Procedure [primary/revision], no. (%)		
1 (primary)	226 (93.8)	
2 (revision)	15 (6.2)	
BMI (kg/m^2^), median [IQR]		
Baseline	27.1 [23.9–30.7]	
BMI (kg/m^2^), baseline, no. (%)		
< 20	6 (2.6)	
20–24.5	65 (28.8)	
25–29.5	87 (38.5)	
30–34.5	49 (21.7)	
35–39.5	14 (6.2)	
≥ 40	5 (2.2)	
Smoking, no. (%)		
Smoker	19 (13.9)	
Nonsmoker	110 (80.3)	
Ex‐smoker	8 (5.8)	
Previous surgery, no. (%)		
Both feet different operation	5 (3.6)	
Both feet same operation	6 (4.3)	
Left foot	26 (18.8)	
Right foot	21 (15.2)	
No operation	80 (58.1)	
Medication, no. (%)		
Aspirin	13 (6.0)	
Direct thrombin inhibitor	1 (0.5)	
Factor xa inhibitor	4 (1.9)	
Low molecular weight heparin	59 (27.7)	
No drug	131 (61.5)	
Other	4 (1.9)	
Warfarin	1 (0.5)	
Patient related outcome measures, median [IQR]		
MOXFQ, pain, baseline	75 [55–85]	
MOXFQ, walking/standing, baseline	71 [50–89]	
MOXFQ, social interaction, baseline	63 [44–81]	
MOXFQ, pain, good, baseline (un‐operated leg)	0.0 [0–15]	
MOXFQ, walk/stand, good, baseline (un‐operated leg)	0.0 [0–18]	
MOXFQ, social interaction, good, baseline (un‐operated leg)	13 [6–25]	
MOXFQ, pain, bad, baseline (operated leg)	65 [50–75]	
MOXFQ, walk/stand, bad, baseline (operated leg)	68 [46–82]	
MOXFQ, social interaction, bad, baseline (operated leg)	50 [31–75]	
NRS pain, baseline	59 [40–70]	
EQ‐5D‐5L‐health‐VAS, baseline	71 [50–85]	
EQ‐5D‐5L, baseline	0.65 [0.42–0.72]	

Abbreviations: BMI, body mass index; MOXFQ, manchester‐oxford foot questionnaire.

Most participants reported having no previous surgery (58.1%). Most participants reported not being prescribed any medication (61.5%), however, 27.7% were prescribed low molecular weight heparin. This reflects data recorded in the registry under the medication field, which primarily captures thromboprophylaxis and not analgesia. Therefore, it should not be inferred that participants did not receive analgesia, as such data were not captured by the registry. The first MTPJ arthrodesis pathway data were classified with distinction of operated and un‐operated legs. The median pre‐operative MOXFQ score for pain related to the operated leg was 65 (50–75). Similarly, pre‐operative median MOXFQ scores for walking/standing related to the operated leg was 68 (46–82). The median pre‐operative NRS score of pain was 59 (40–70), whereas median pre‐operative EQ‐5D‐5L health VAS was 71 (50–85). Additionally, the median pre‐operative EQ‐5D‐5L score was 0.65 (0.42–0.72).

### 5.2 Data Completeness

Data completeness was evaluated for demographic, clinical and PROM data items, reported as a proportion of the total population (*n* = 459) (Table 2). At baseline, data for participants' sex were available for 99% of the study population, BMI for 49% and medication related information for 46%. Surgical procedures were recorded for just over 52%. There was scant information concerning participants' mobility, procedures and adverse events. Record of the type of surgery and surgical procedures were variably recorded, for example, surgical approach for 21.8% and fixation 51.4%, with revision recorded well (52.1%). Only 1.9% adverse events were recorded; however, due to substantial missing data, this figure may not accurately reflect the true incidence of adverse events.

**TABLE 2:** Completeness of data in the First MTPJ Arthrodesis pathway.

Data item	Completed record (*n*)	Missing (*n*)	Completion rate (%)
BMI			
Baseline	226	233	49.2
6 months	30	429	6.5
12 months	5	454	1.1
2 years	1	458	0.2
All types of surgery	49	410	10.7
Initial weight bearing	231	228	50.5
Partial weight bearing	87	372	19.0
Assistance with partial weight bearing	94	365	20.6
Other partial weight bearing	6	453	1.3
Assistance with full weight bearing	121	338	26.4
Other assistance with full weight bearing	1	446	2.8
Duration weight bearing/week	195	264	42.5
Other weight bearing status	4	455	09
Second degree weight bearing	88	371	19.2
Second degree partial weight bearing	3	456	0.7
Assistance/full weight bearing	82	377	17.9
Assistance 2^nd^ degree weight bearing	82	377	17.9
Other 2^nd^ degree full weight bearing	4	455	0.9
Duration 2^nd^ degree full weight bearing	12	447	2.6
3^rd^ degree full weight bearing	3	456	0.7
Assistance 3^rd^ degree full weight bearing	3	456	0.7
Adverse events	5	454	1.9
Further adverse events recorded	4	455	0.8
Approach	100	359	21.8
Revision	239	220	52.1
Insertion	17	442	3.3
Fixation	237	222	51.4
Screws	65	394	13.8
Types of screws	158	301	34.1
Procedure	93	366	20.5
Further surgery	222	237	48.4
Plate	182	277	39.7

Abbreviation: BMI, body mass index.

### 5.3 Descriptive Analysis of PROMs—Pre‐ and Post‐Clinical Outcomes

Tables 3 and 4 describe the pre‐ and post‐operative clinical outcome scores, and data completeness. Data entry completeness varied across different time points and outcome measures. For MOXFQ scores for the operated leg, the completion rate was 52.5% for pain and walking/standing at baseline (27.2% at 6 months and 15.7% at 12 months), and 50.3% for social interaction at baseline (26.4% at 6 months and 15.0% at 12 months). Completion rates for the Numeric Rating Scale (NRS) for pain ranged from 54.2% at baseline to 14.2% at 12 months indicating considerable variability in data collection success. Likewise, completion rates for the EQ‐5D‐5L Health‐VAS and EQ‐5D‐5L measures varied from 70.8% at baseline to 17.9% at 12 months, demonstrating differences in data entry success over time. Improvements in scores exceeded the Minimal Clinically Important Difference (MCID), highlighting the clinical significance of findings. The improvements in the MOXFQ domains of walking/standing, pain, and social interaction exceeded the MCIDs of 16, 12 and 24 respectively, indicating the clinical significance of results [18]. The MCID for NRS pain on a 0–100 scale is typically between 10 and 20 points, suggesting a meaningful change in pain levels [19, 20]. Previous research has reported MCIDs for EQ‐5D‐5L in musculoskeletal participants, such as 0.03 [21] and 0.081 [22] for low back pain.

**TABLE 3:** Data completeness/missingness of MOXFQ—operated/un‐operated leg.

Phase III—operated/un‐operated leg [*n* = 459]	Completed record (*n*)	Missing (*n*)	Completion rate (%)
MOXFQ—bilateral unoperated leg—(pain and walking/standing)^a^			
Baseline	73	386	15.9
6 months	43	416	9.4
12 months	23	436	5.0
2 years	2	457	0.4
MOXFQ—bilateral unoperated leg—(social interaction)^a^			
Baseline	63	396	13.7
6 months	39	420	8.5
12 months	22	437	4.8
2 years	2	457	0.4
MOXFQ—bilateral operated leg—(pain and walking/standing)^a^			
Baseline	241	218	52.5
6 months	125	334	27.2
12 months	72	387	15.7
2 years	2	457	0.4
MOXFQ—bilateral operated leg (social interaction)^a^			
Baseline	231	228	50.3
6 months	121	338	26.4
12 months	69	390	15.0
2 years	2	457	0.4

^a^No observation recorded at 5‐year interval.

**TABLE 4:** Data completeness/missingness of NRS (pain)/EQ‐5D‐5L‐Health‐VAS/EQ‐5D‐5L.

NRS (pain)^a^ (*n* = 459)	Completed record (*n*)	Missing (*n*)	Completion rate (%)
Baseline	249	210	54.2
6, months	120	339	26.1
12, months	65	394	14.2
EQ‐5D‐5L‐health‐VAS^b^			
Baseline	325	134	70.8
6, months	157	302	34.2
12, months	82	377	17.9
2, years	3	456	0.7
EQ‐5D‐5L^b^			
Baseline	325	134	70.8
6, months	157	302	34.2
12, months	82	377	17.9
2, years	3	456	0.7

^a^No observation recorded at 2‐ and 5‐year intervals.

^b^No observation recorded at 5‐year interval.

### 5.4 Evaluation of Clinical Outcomes Following First MTPJ Arthrodesis

#### 5.4.1 MOX‐FQ—Pain

**FIGURE 1:**
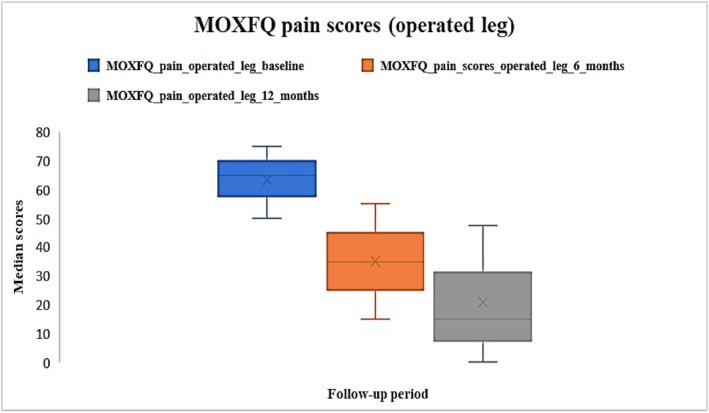
Median MOX‐FQ pain scores for the operated leg at baseline, 6 months and 12 months post‐operatively.

Median pre‐operative MOX‐FQ score for pain for the operated leg was 65 [IQR: 50–75], and at 6 and 12 months were 35 [IQR: 15–55] and 15 [IQR: 0–47.5] respectively. Post‐operative scores were improved compared to baseline (Figure 1). Missing data were approximately 84% at baseline, 91% at 6 months and 95% at 12 months.

#### 5.4.2 MOX‐FQ Walking/Standing

**FIGURE 2:**
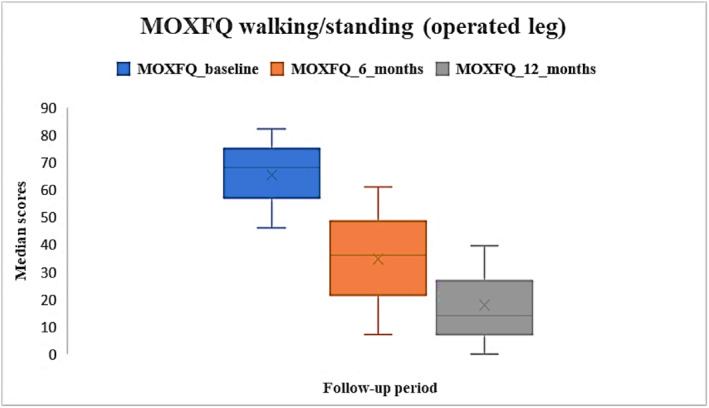
Median MOX‐FQ walking/standing scores for the operated leg at baseline, 6 months and 12 months post‐operatively.

Median pre‐operative MOX‐FQ score for walking/standing for the operated leg was 68 [IQR: 46–82], and at 6 and 12 months were 36 [IQR: 7.0–61] and 14 [IQR: 0–39.5] respectively. Post‐operative scores were improved compared to baseline (Figure 2). Missing data were approximately 47% at baseline and 73% at 6 months and 84% at 12 months intervals.

#### 5.4.3 MOX‐FQ Social Interaction

**FIGURE 3:**
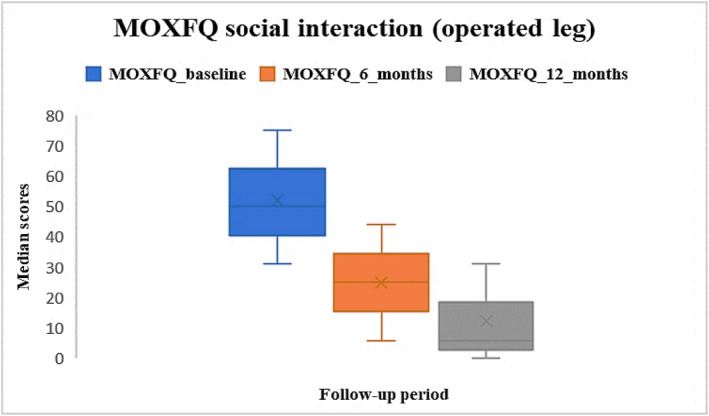
Median MOX‐FQ social interaction scores for the operated leg at baseline, 6 months and 12 months post‐operatively.

Median pre‐operative MOX‐FQ score for social interaction for the operated leg was 50 [IQR: 31–75], and at 6 and 12 months were 25 [IQR: 6–44] and 6 [IQR: 0–31] respectively. Post‐operative scores were improved compared to baseline (Figure 3). Missing data were approximately 50% at baseline, 74% at 6 months and 85% at 12 months.

### 5.5 NRS (Pain) and EQ‐5D‐5L‐Health‐VAS

**FIGURE 4:**
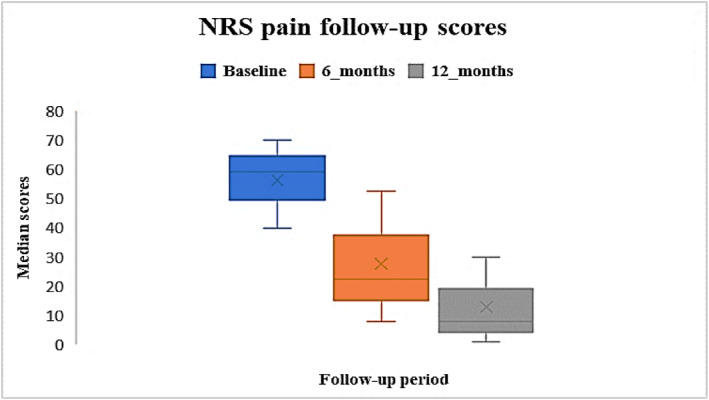
Median NRS (pain) scores at baseline, 6 months and 12 months post‐operatively.

**FIGURE 5:**
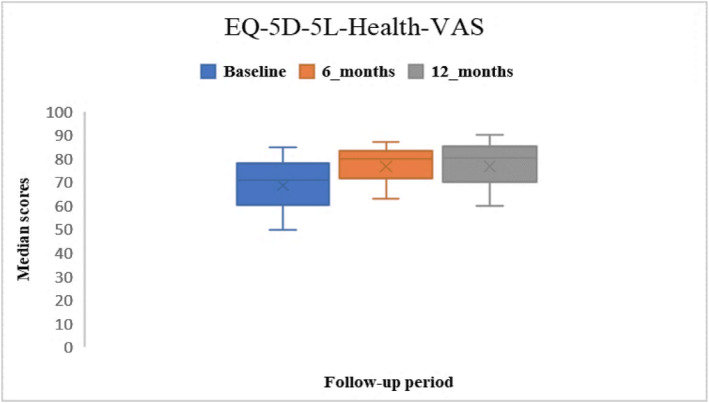
Median EQ‐5D‐5L Health VAS scores at baseline, 6 months and 12 months post‐operatively.

Pre‐operative median NRS (pain) was 59 [IQR: 40–70], and at 6 and 12 months were 22.5 [IQR: 8–52.5] and 8 [IQR: 1–30] respectively. Pre‐operative median EQ‐5D‐5L‐Health‐VAS was 71 [IQR: 50–85], and at 6 and 12 months were 80 [IQR: 63–87] and 80.5 [IQR: 60–90] respectively. Post‐operative scores were improved compared to baseline (Figures 4 and 5). NRS pain data were missing for 46% at baseline, at 6 months this was 74% and reduced to 86% at 12 months. EQ‐5D‐5L‐Health VAS data were missing for 31% at baseline, and at 6 and 12 months these were 66% and 82% respectively.

### 5.6 EQ‐5D‐5L

**FIGURE 6:**
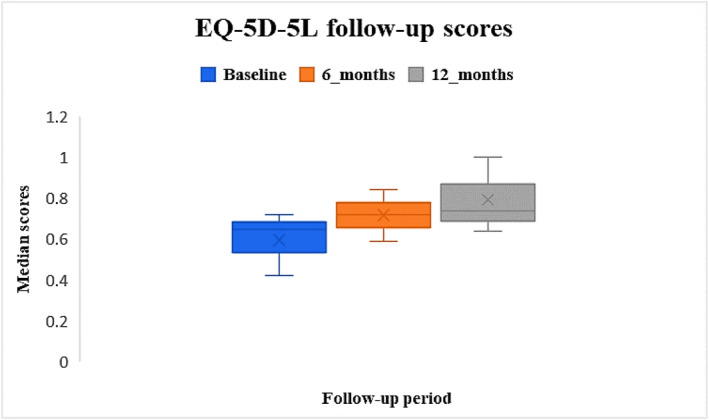
Median EQ‐5D‐5L scores at baseline, 6 months and 12 months post‐operatively.

Pre‐operative median EQ‐5D‐5L was 0.65 [IQR: 0.42–0.72], and at 6 and 12 months were 0.72 [IQR: 0.59–0.84] and 0.74 [IQR: 0.64–1.0] respectively. Post‐operative scores were improved compared to baseline (Figure 6). Missing data at baseline were similar to that of EQ‐5D‐5L‐Health‐VAS at baseline of 31%, and 66% and 82% at 6 and 12 month.

### 5.7 Risk Groups

The median [IQR] of good versus poor outcome with associated p values for all the study PROMs are presented in Table 5. These estimates should be treated with caution due to the high rates of missing data. Most participants show a reduction in pain intensity, improved walking/standing ability, and social interaction by 12 months after surgery. There is a statistically significant difference in the median of good outcome versus poor outcome in the MOXFQ PROM median scores (*p* = 0.001). NRS and EQ‐5D‐5L also show a statistically significant difference in median scores (*p* = 0.001) by 12 months following surgery. Only EQ‐5D‐5L‐Health VAS did not show a statistically significant association between the median scores (poor vs. good outcome).

**TABLE 5:** MOXFQ post‐operative pain outcome (good vs. poor).

Outcome	Number	Median [IQR]	p‐value [good vs. poor outcome]
MOXFQ Pain (operated leg)			
Good	42	10 [IQR: 0–20]	< 0.001
Poor	1		
MOXFQ walking/standing (operated leg)			
Good	40	5.5 [IQR: 0, 21]	< 0.001
Poor	4	67.5 [IQR: 49.7, 78.2]	
MOXFQ social/interaction (operated leg)			
Good	38	0 [IQR: 0, 19]	< 0.001
Poor	4	91 [IQR: 67.5, 94]	
NRS			
Good	36	5 [IQR: 1, 31]	< 0.001
Poor	4	64 [IQR: 52.5, 68.5]	
EQ‐5D‐5L‐health VAS			
Good	32	61 [IQR: 50, 80]	0.891
Poor	27	90 [IQR: 71, 93]	
EQ‐5D‐5L			
Good	51	0.8 [IQR: 0.7, 1.0]	< 0.001
Poor	10	0.5 [IQR: 0.4, 0.6]	

## 6 Discussion

This is the first study to analyse data collected regarding first MTPJ arthrodesis through the BOFAS registry that was created to enable tracking of participant outcomes to allow for comparison locally and nationally. BOFAS established the registry in 2014. Currently the registry holds over 5000 records with an increase year on year. To enable the registry to fulfil its aim the completeness and consistency of the data captured is essential. Although the BOFAS registry cannot establish causal effectiveness due to its observational design, it provides valuable evidence of treatment outcomes by systematically capturing pre‐ and post‐operative PROMs. These data show significant improvements in pain, function, and quality of life, allowing for benchmarking across institutions and over time. However, to definitively establish the effectiveness of treatment, randomised controlled trial (RCT) data would be required. The importance of PROMs in the evaluation of foot and ankle surgery has been demonstrated for hallux valgus surgery by Baumhauer et al. (2013) [23] also illustrating that the outcome regarded by participants differs from those evaluated by doctors indicating a degree of inconsistency in expectations between participants and doctors. This divergence highlights the value of incorporating participant perspectives into outcome evaluation, as it ensures that surgical success is not solely defined by clinical or radiographic criteria, but also by improvements in function, pain, and quality of life as perceived by the participant. The BOFAS registry, by collecting PROMs directly from participants, allows clinicians and service providers to better align care with what matters most to participants, ultimately enhancing shared decision‐making and patient‐centred care. By capturing PROMs directly from participants at several intervals, the registry provides valuable insight into participant expectations and how these evolve over time, supporting a more informed and responsive care approach.

### 6.1 Completeness of Data/Capture

Data completeness was evaluated, and data items reported as a proportion of the total population, defined as all 459 participants who had baseline PROMs data available and were included in the study cohort. The level of demographic and clinical data at baseline were adequately recorded. There were some items that were more prone to incorrect entries, for, example, BMI where some extreme values were observed. It is important that variables such as BMI are recorded accurately as omitting such variables may limit the accuracy of the analyses where BMI should be included as a covariate. A potential explanation as to why completeness rates are problematic in National Joint Registries [24] is their reliance on data input at local level, which is subject to variation across hospitals. The completion rate for PROMs at baseline was adequate; for example, MOXFQ pain and walking/standing 52.5%. Completion rates then declined rapidly for 6 months (12.7%) and 12 months (4.8%) intervals. One of the main reasons that PROMs are poorly recorded at 6 and 12 months may be because the PROMs require participants to report on their own health status without prompt or encouragement/support from healthcare professionals. Without mandatory completion, the BOFAS registry relies on data input without formal resource funding, thus ensuring your own data are complete as a surgeon is done outside of normal working practice. Furthermore, participants may find this process time consuming and labour intensive. There are many factors that could potentially influence response rates. A study by Palmen and colleagues (2016) [25], comparing three methods of administration (mail, telephone and email) in 73 participants following hallux valgus surgery, found that email had the lowest completion rate (33%), and conversely traditional mail had the highest (88%), with telephone also high at (70%). NHS England currently mandates the collection of PROMS for hip and knee replacement, mandated by the Department for health in 2009. The BOFAS registry is not currently mandated, and this may affect the way resources are allocated at the organisation level. The present study shows a completion rate steadily decreasing at 6 months (30%) and 12 months (16%), raising the question as to whether requesting participants to complete PROMs electronically and contacting participants via email/telephone to complete PROMs are effective strategies. Widnall et al. (2020), reported a 97% completion rate of pain scores when using an automated text message to parents of children with distal radius fractures [4] which may be a useful development for BOFAS. The registry helps assess variation in performance by allowing comparisons of participant outcomes across different surgeons and institutions, identifying outliers and promoting standardisation of care practices. This benchmarking facilitates continuous quality improvement and supports clinical audit.

Data completeness may have implications for the consistency and usefulness of the results derived from registry data because these results are inherently dependent on the quality and completeness of data being entered into the registry. Further development of robust processes in data collection and checking through real time monitoring of the recorded data complemented by internal audit of data quality is crucial to ensure the integrity of the database and reporting capability. Further coding of data might be a way to increase accuracy of data entry. Coding data requires less time to enter and reduces duplication of data items entered; for example, adverse events could be coded rather than entering as individual items. Coding also helps in appropriately sorting data during the data transformation process and can save valuable memory/storage space. Amplitude, which host the registry, has implemented some changes to allow better data collection and reduce missing data. The registry supports patient‐centred care by capturing what matters most to participant's pain relief, mobility, and quality of life at multiple time points. This enables clinicians to tailor treatment decisions and follow‐up care to individual needs and expectations. As more surgeons join the registry, the number of records will increase, which has the potential to enhance data reliability, provided that data completeness and quality are simultaneously improved through robust governance, mandatory fields, and consistent clinician engagement. Without such measures, increasing participation alone may not address existing limitations in data validity.

### 6.2 Evaluation of PROMs Pre‐ and Post‐Surgery

The First MTPJ Pathway reports pre‐ and post‐PROMs for primary and revision procedures. The system is designed to record PROMs electronically, as this allows the measuring and comparing of factors that participants find important during their recovery. Symptom burden, quality of life and satisfaction with care are recorded by participants in real time allowing capture of these essential outcome measurements ‘in the moment’ which cannot be recreated accurately through retrospection. For this reason, routine, systematic, and longitudinal collection of PROMs should be a standard of clinical practice [26]. Unlike the comprehensive PROM follow‐up achieved for hip and knee arthroplasty with mandated collection of PROMs, and dedicated PROM teams ensure high completion rates, there is a noticeable lack of similar focus and resources for foot and ankle procedures, highlighting a significant discrepancy in participant outcome tracking. The comparison of pre‐ and post‐surgery scores illustrated improvements in the MOX‐FQ score for pain for the operated leg, from a median of 65 pre‐surgery to 35 at 6 months and 15 at 12 months. The same change was illustrated by the NRS pain scores, from a median of 59 pre‐surgery to 22.5 at 6 months and 8 at 12 months. These findings illustrate that the surgery contributed considerable improvements in pain. Interestingly, for the un‐operated leg the data demonstrated worsening MOX‐FQ scores for pain from a median of 0 at baseline to 10 at 6 months and 5 at 12 months; perhaps reflecting the impact of the pathology on the opposite leg that is required to compensate to enable continued function. The same pattern of improvement was illustrated for the MOX‐FQ scores for walking/standing and social interaction. The reductions in MOXFQ median scores by 12 months following surgery were statistically significant (*p* = 0.001), illustrating that most participants demonstrated reductions in pain intensity, improved walking/standing ability, and social interaction by 12 months after surgery. The comparison of pre‐ and post‐surgery scores illustrated improvements in EQ‐5D‐5L‐Health‐VAS, from a median of 71 pre‐surgery to 80 and 80.5 at 6 at 12 months; and improvements in EQ‐5D‐5L from a median of 0.65 pre‐surgery to 0.72 at 6 months and 0.74 at 12 months. These finding illustrated that quality of life improved in the first 6 months following surgery but that no further improvement was demonstrated after 6 months. However, this interpretation should be treated with caution given the extent of missing data at 12 months, which may have limited the ability to detect a clinically meaningful change beyond the 6‐month mark.

### 6.3 Post‐Operative (Poor vs. Good) Outcome Following Surgery

There was a statistically significant difference in the median of good outcome versus poor outcome in the MOXFQ NRS and EQ‐5D‐5L scores (*p* = 0.001), illustrating the significant differences between participants experiencing a good versus poor outcome and highlighting the importance of being able to distinguish between these 2 groups of participants to select participants for surgery/provide rehabilitation. Only EQ‐5D‐5L‐Health VAS did not show a statistically significant association between the median scores (poor vs. good outcome). However, these estimates should be treated with caution due to high rates of missing data. Overall, the clinical outcome data suggest significant improvements following surgical intervention. These findings illustrate potential effectiveness of surgery on all outcomes following first MTPJ arthrodesis (MOX‐FQ scores for pain, walking/standing and social interaction; NRS pain; EQ‐5D‐5L; and EQ‐5D‐5L‐Health‐VAS), that merit evaluation in a clinical trial. Access to anonymised registry data for future research is available upon formal application to BOFAS, subject to governance approvals, making it a valuable resource for ongoing clinical and academic inquiry. Furthermore, the BOFAS registry enables comparison of outcomes at both local and national levels, providing individual surgeons and institutions with insights into their performance relative to peers, thereby fostering reflective practice and quality improvement.

### 6.4 Comparison With Other Registries

The BOFAS registry, while providing valuable patient‐reported outcome data for first MTPJ fusion, faces challenges common to many clinical registries, including incomplete data capture and voluntary participation. Comparable national registries, such as the National Joint Registry (NJR) in the UK, which monitors hip and knee arthroplasty outcomes, have demonstrated the benefits of mandatory reporting and standardised data collection protocols to enhance data completeness and reliability [30]. The NJR's integration with clinical workflows and robust governance has enabled it to influence national policy and improve patient outcomes effectively. Similarly, the Australian Orthopaedic Association National Joint Replacement Registry has shown that continuous data quality improvement and clinician engagement are critical to registry success [31]. By contrast, the BOFAS registry's current voluntary design and data loss limit its impact. Learning from established registries, implementing mandatory minimum datasets, and embedding data collection within routine clinical practice could strengthen BOFAS's utility as a national audit tool.

### 6.5 Strengths and Limitations

The strength of this study is its novel analysis of the BOFAS registry data and inclusion of 459 participants. However, there were some limitations. For all analyses, wide IQRs illustrated variability across participants on all measures, and analyses were limited by missing data, particularly at 6 and 12 months follow‐up time points. A further limitation is that direct comparison with national databases (e.g., UK HES database) was not possible because of a lack of comparability. A key limitation of this study is that the BOFAS registry data are collected on a voluntary basis and therefore may not fully represent all MTPJ arthrodesis procedures performed across the UK during the study period. Because the registry reflects data submitted by only a subset of participating surgeons and centres, this may introduce selection bias and limit the generalisability of the findings. Furthermore, while automated follow‐up provides a streamlined approach to record PROMS, this study has revealed a notable drop in follow‐up rates over time, prompting doubts about its efficacy. It may be beneficial to explore the cost‐effectiveness of alternative follow‐up methods. For instance, employing a designated BOFAS Registry PROM collector to send letters or call participants might improve completion rates. It is also worth noting the burden of the multiple data fields within the registry as there is also poor data entry at baseline. Perhaps redefining what constitutes critical baseline and follow‐up data could streamline the process, making it less of a burden for completion. Additionally, the very high proportion of female participants (98.9%) is notable and may not reflect the true gender distribution of participants undergoing this procedure in broader clinical practice, warranting caution in generalising findings and consideration of potential selection bias. Furthermore, the inability to compare baseline characteristics between responders and nonresponders due to limited data on nonresponders restricts assessment of potential attrition bias, which may impact the generalisability of the findings. Additionally, as this was an uncontrolled observational study, the outcomes reported may be influenced by factors such as the Hawthorne effect or regression to the mean, which cannot be ruled out. These inherent limitations should be considered when interpreting the findings.

## 7 Conclusion

The BOFAS registry provides a framework for the registration and quality monitoring of foot and ankle surgery. The registry has shown that first MTPJ arthrodesis improves outcomes, illustrating the benefit of surgery on all outcomes, including reductions in pain intensity, and improvements in walking/standing ability, social interaction and overall quality of life that are clinically significant. These findings should be interpreted with caution due to methodological limitations, including potential selection bias, reporting bias and high levels of missing follow‐up data. However, limitations in data completeness at all timepoints may impact the consistency and usefulness of the results derived from registry data. This necessitates further development of robust processes in data collection, checking, and internal audit of data quality. Although this analysis highlights these challenges, improving data quality could enhance the registry's potential for nationwide quality assessment of foot and ankle surgery, including participants on the first MTPJ arthrodesis pathway. These findings demonstrate the potential effectiveness of surgery on various outcomes following first MTPJ arthrodesis, which merits evaluation in a clinical trial. Screenshots of the patient and clinician portals, which form part of the registry's infrastructure, can be found in Appendix 1 for reference.

## 8 Summary

- Most participants demonstrated reductions in pain intensity, improved walking/standing ability, social interaction and quality of life following first MTPJ arthrodesis.
- These findings illustrate potential effectiveness of the surgery on all outcomes.
- As the quality of the data recorded improves better, more robust associations can be inferred
- These analyses have demonstrated the potential for the BOFAS registry becoming a valuable clinical tool as data quality improves. However, this potential is contingent on implementing strategies to reduce data loss and improve data completeness.

## Author Contributions

**Ferozkhan Jadhakhan:** conceptualisation, data curation, formal analysis, investigation, methodology, project administration, writing – original draft, writing – review and editing. **Makwana Nilesh:** conceptualisation, methodology, writing – review and editing. **Mason Lyndon:** conceptualisation, methodology, writing – review and editing. **Halliwell Paul:** conceptualisation, methodology, writing – review and editing. **Rushton Alison:** conceptualisation, methodology, writing – original draft, writing – review and editing.

## Ethics Statement

This study was approved by the University of Birmingham Ethics Committee (ERN_191274AP2).

## Consent

The authors have nothing to report.

## Conflicts of Interest

The authors declare no conflicts of interest.

## Data Availability

The authors have nothing to report.
